# Knowledge-Aided Doppler Beam Sharpening Super-Resolution Imaging by Exploiting the Spatial Continuity Information

**DOI:** 10.3390/s19081920

**Published:** 2019-04-23

**Authors:** Hongmeng Chen, Zeyu Wang, Jing Liu, Xiaoli Yi, Hanwei Sun, Heqiang Mu, Ming Li, Yaobing Lu

**Affiliations:** 1 Beijing Institute of Radio Measurement, Beijing 100854, China; chenhongmeng123@163.com (H.C.); jingliu_sp2306@163.com (J.L.); 13466798179@163.com (X.Y.); 13488682636@126.com (H.S.); 13611258792@163.com (H.M.); 2 China Academy of Electronics and Information Technology, China Electronic Technology Group Corporation, Beijing 100846, China; beidou13579@163.com; 3 National Laboratory of Radar Signal Processing, Xidian University, Xi’an 710071, China; liming@xidian.edu.cn

**Keywords:** wide-area surveillance, super-resolution, Doppler beam sharpening

## Abstract

This paper deals with the problem of high cross-range resolution Doppler beam sharpening (DBS) imaging for airborne wide-area surveillance (WAS) radar under short dwell time situations. A knowledge-aided DBS (KA-DBS) imaging algorithm is proposed. In the proposed KA-DBS framework, the DBS imaging model for WAS radar is constructed and the cross-range resolution is analyzed. Since the radar illuminates the imaging scene continuously through the scanning movement of the antenna, there is strong spatial coherence between adjacent pulses. Based on this fact, forward and backward pulse information can be predicted, and the equivalent number of pulses in each coherent processing interval (CPI) will be doubled based on the autoregressive (AR) technique by taking advantage of the spatial continuity property of echoes. Finally, the predicted forward and backward pulses are utilized to merge with the initial pulses, then the newly merged pulses in each CPI are utilized to perform the DBS imaging. Since the number of newly merged pulses in KA-DBS is twice larger than that in the conventional DBS algorithm with the same dwell time, the cross-range resolution in the proposed KA-DBS algorithm can be improved by a factor of two. The imaging performance assessment conducted by resorting to real airborne data set, has verified the effectiveness of the proposed algorithm.

## 1. Introduction

Airborne or spaceborne wide-area surveillance (WAS) radar [1,2] can acquire a wide-area surveillance scene at a very short time, which is usually accomplished by steering the antenna beam from one azimuth angle to another. In the scanning movement of the antenna, the dwell time at each azimuth angle is very short to guarantee a high revisit radio. Accordingly, airborne or spaceborne WAS radar is widely applied in civilian and military fields [3,4,5,6,7], and Doppler beam sharpening (DBS) technique is a very effective tool to accomplish the WAS ability [8,9,10,11]. However, the large surveillance region in WAS radar is at the expense of low cross-range resolution. The low cross-range resolution limits its further application. Therefore, it is definitely essential to study the high cross-range resolution further for airborne or spaceborne WAS radar in short dwell time situations.

Several researchers have studied this issue of WAS imaging in previous works, Scan-synthetic aperture radar (Scan-SAR) [1,12,13] can acquire a wide-region compared with conventional TOPs SAR mode [14,15], strip SAR mode [16,17,18] and spotlight SAR mode [19], and it is an effective means for WAS imaging. However, the synthetic time is usually as large as 1~10 s, which limits the high revisit ratio. Since DBS is the non-focused form of SAR [1,8,9,10,11], the imaging time for DBS is usually as small as 0.05~0.1 s, the revisit ratio is very high. Therefore, our attention in this paper is mainly paid to the DBS imaging. For DBS imaging, Fourier transform (FT) [1,8,9], Relax [10], APES [11] are used to increase the cross-range resolution. However, the performance of these existing methods is generally not satisfactory in the engineering applications.

In this paper, we propose an efficient DBS cross-range resolution enhancement architecture, namely knowledge-aided DBS (i.e., KA-DBS), to increase the DBS imaging performance. For the airborne WAS radar, the antenna usually works in a scanning mode, where the antenna illuminates the surveillance region continuously by steering the antenna beam from one azimuth viewing angle to another. Therefore, the echoes reflected from the scatterers on the ground may be coherent in the space. The space coherence property means that more spatial information about the echoes may be mined if proper means are used. Based on this fact, the knowledge of the spatial coherence property is fully exploited in KA-DBS. And then, the spatial continuity model of the radar echo is constructed. In order to well estimate the pulses information outside the observed coherent processing interval (CPI), the forward prediction pulses and the backward prediction pulses are estimated based on the autoregressive (AR) technique [20,21,22], respectively. Accordingly, the number of pulses at one azimuth angle can be equivalently increased by merging the forward prediction pulses and the backward prediction pulses with the original pulses. Finally, the “merged pulses” are utilized to perform the DBS imaging. The number of “merged pulses” in the proposed KA-DBS algorithm is twice larger than that in the conventional DBS algorithm with the same dwell time. Therefore, the cross-range resolution in KA-DBS is doubled compared with the conventional DBS imaging algorithm. Real-data results show that the proposed algorithm performs well with short dwell time.

The rest of this paper is organized as follows: in Section 2, the DBS architecture is discussed. In Section 3, we introduce the novel KA-DBS algorithm in detail. The performance of the proposed algorithm is verified by real measured data in Section 4. Finally, some conclusions are given in Section 5.

## 2. DBS Imaging Model

**Figure 1:**
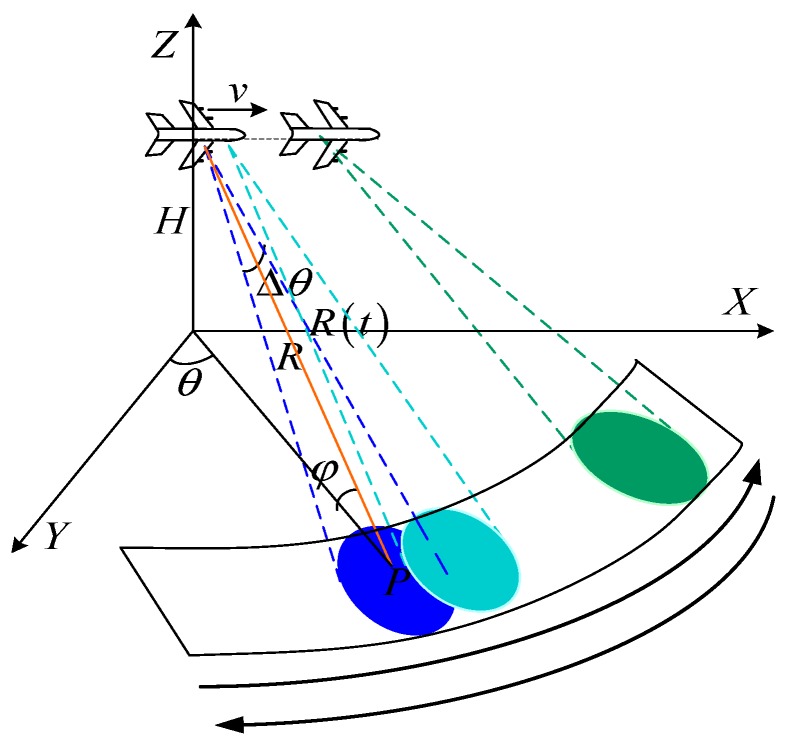
Geometry of DBS imaging.

For airborne WAS radar system, the radar dwells in a particular beam position continuously with a set of coherent processing intervals (CPIs). The surveillance region is searched by sequentially looking in all azimuth angles, the working mode of the WAS radar is illustrated in Figure 1.

Suppose that the aircraft flies along to the *X*-axis with velocity *v*, and with the flight altitude, *H*, and the initial slant range between the target, *R*_0_. The azimuth angle and the elevation angle are *θ* and *ϕ*, respectively. It is assumed that a linear frequency-modulated (LFM) signal is transmitted, and it can be written as:

(1) $$s\left(\tau \right)=\mathrm{rect}\left(\frac{\tau }{T_{p}}\right)\exp\left[j2\pi \left(f_{c}\tau +\frac{\gamma }{2}\tau^{2}\right)\right]$$

where *τ* denotes the fast time, *T_p_* denotes the pulse width, *f_c_* is the carrier frequency of the transmitted signal, and rect(·) stands for the unit rectangular function. *γ* is the chirp rate. The echoed signal reflected from a point target can be expressed as:

(2) $$s(\tau ,t)=\sigma \cdot \mathrm{rect}\left(\frac{\tau -\frac{2R(t)}{c}}{T_{p}}\right)\exp\left\{j2\pi \left[f_{c}\left(\tau -\frac{2R(t)}{c}\right)+\frac{\gamma }{2}{\left(\tau -\frac{2R(t)}{c}\right)}^{2}\right]\right\}$$

where *t* denotes the slow time, *R*(*t*) is the instantaneous slant range history from the radar to the point target at time *t*. *σ* is the radar cross-section (RCS) of the target, and *c* is the velocity of light.

According to the DBS geometry illustrated in Figure 1, the instantaneous slant range history between the target and the radar can be written as:

(3) $$R\left(t\right)=\sqrt{R_{0}^{2}+{\left(vt\right)}^{2}-2R_{0}vt\sin\theta \cos\varphi }$$

Equation (3) can be expanded into a Taylor series, and it can be expressed as:

(4) $$R\left(t\right)=R_{0}-vt\sin\theta \cos\varphi +\frac{{\left(vt\right)}^{2}\left(1-\sin^{2}\theta \cos^{2}\varphi \right)}{2R_{0}}+O\left(t^{3}\right)$$

Since the dwell time in each fixed azimuth viewing angle is very short, the value of the airplane travels *vt* is far less the slant range *R*_0_ (i.e., *vt* □ *R*_0_), then the instantaneous slant range history can be approximately expressed as:

(5) $$R\left(t\right)\approx R_{0}-vt\sin\theta \cos\varphi$$

The Doppler centroid can be estimated by the follows:

(6) $$f_{d}=-\frac{2}{\lambda }\frac{dR\left(t\right)}{dt}=\frac{2v\sin\theta \cos\varphi }{\lambda }$$

It can be known that different azimuth angles corresponds to different Doppler frequencies. Therefore, the problem of distinguishing different scatterers at different azimuth angles can be transformed into the problem of distinguishing different scatterers at different Doppler frequencies. Suppose a scatterer is located at the azimuth angle *θ*_0_, and the azimuth angle *θ*_0_ is also the center of the antenna beam. The boundaries of the antenna beam can be denoted as *θ*_0_ – Δ*θ*/2 and *θ*_0_ + Δ*θ*/2, which correspond to the Doppler frequencies *f_dh_* and *f_dl_*, respectively. Δ*θ* is the 3 dB width of the radar beam. The Doppler bandwidth of the scatterer can be derived as:

(7) $$\Delta f_{d}=\left|f_{dh}-f_{dl}\right|=\left|f_{d}\left(\theta_{0}-\frac{\Delta \theta }{2}\right)-f_{d}\left(\theta_{0}+\frac{\Delta \theta }{2}\right)\right|=\frac{2v\cos\theta_{0}\cos\varphi }{\lambda }\Delta \theta$$

The Doppler bandwidth illustrated in Equation (7) is the frequency excursion experienced by the scatterer during the dwell time in which the scatter is illuminated by the 3 dB width of the antenna. Assuming that pulse compression and range migration correction [23] are performed, then the echoed signal can be written as:

(8) $$s(\tau ,t)=\sigma \cdot \mathrm{rect}\left(\frac{t}{T_{a}}\right)\sin c\left[B\left(\tau -\frac{2R_{0}}{c}\right)\right]\exp\left(-j4\pi \frac{R(t)}{\lambda }\right)$$

where *T_a_* = *N_a_T_r_*, is defined as the CPI in one look direction, and *T_r_* is the pulse repetition interval (PRI). *N_a_* is the coherent pulse number in one CPI. Assuming that there are *K* scattering centers in one range cell, and fast Fourier transform (FFT) is performed to get the DBS imaging result [1,8,9]:

(9) $$S\left(\tau ,f\right)=\sin c\left[B\left(\tau -\frac{2R_{0}}{c}\right)\right]\mathrm{sinc}\left[T_{a}\left(f-f_{k}\right)\right]$$

From Equation (9), it can be seen that the Doppler resolution in the conventional FFT-based method is approximately determined by:

(10) $$\delta f_{d}=1/T_{a}$$

where *δf_d_* is the Doppler resolution.

One important parameter is the sharpening ratio of a DBS image, *K_a_*, as the ratio of the Doppler bandwidth to the Doppler resolution, is given by [1]:

(11) $$K_{a}=\Delta f_{d}/\delta f_{d}=\Delta f_{d}T_{a}$$

For DBS imaging, the velocity of the aircraft and the antenna beam width are always fixed, which makes the Doppler bandwidth is fixed at the given azimuth viewing angle. Since the CPI in one look direction satisfies *T_a_* = *N_a_T_r_*, then Equation (11) can be rewritten as:

(12) $$K_{a}=\Delta f_{d}N_{a}T_{r}$$

From Equation (11), we can know that increasing the Doppler bandwidth, the coherent pulse number as well as the pulse repetition interval is a useful way to increase the sharpening ratio *K_a_*. However, the sharpening ratio *K_a_* cannot be increased infinitely.

**Figure 2:**
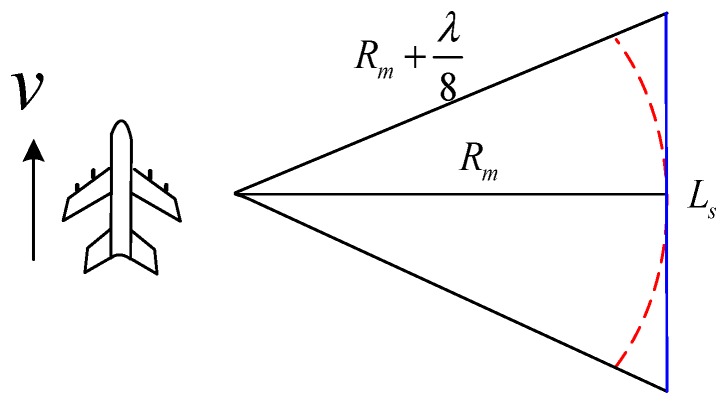
Illustration of the maximum aperture length in DBS imaging.

To estimate the maximum value of the sharpening ratio *K_a_*, we should derive the maximum aperture length in DBS imaging. Assuming that the mean slant range in Figure 2 is *R_m_*, then we can get the maximum effective aperture length *L_s_* in one CPI [1], which satisfies:

(13) $$\sqrt{{R_{m}}^{2}+{\left(\frac{L_{s}}{2}\right)}^{2}}-R_{m}\leq \frac{\lambda }{8}$$

After some mathematical derivation, the maximum aperture length in DBS imaging is given by:

(14) $$L_{s}\leq \sqrt{R_{m}\lambda }$$

and the maximum number of pulses in one CPI can be calculated as:

(15) $$N_{\max}=\frac{\sqrt{R_{m}\lambda }}{vt_{r}}$$

Therefore, the sharpening ratio has the upper boundary:

(16) $$K_{a}\leq \Delta f_{d}N_{\max}T_{r}$$

In order to guarantee the wide-area surveillance ability and high revisit ratio, the number of pulses in one CPI is bounded by the maximum value *N*_max_. The conflict between short CPI and high resolution in cross-range motivates the study of super-resolution approaches for DBS imaging.

Inspecting Equation (16), we can find that increasing the equivalent number of pulses at each CPI in one look direction may be effective to improve the cross-range resolution. In the following section, we will consider an alternative strategy to increase the sharpening ratio *K_a_* to improve the DBS imaging performance.

## 3. Knowledge-Aided DBS Super-Resolution Imaging Algorithm

### 3.1. Spatial Continuity Property of the Echoed Signal

When the antenna of the airborne WAS radar scans the surveillance region, the radar illuminates the imaging scene continuously through the scanning movement of the antenna beam. Since the antenna beam is steered from one azimuth viewing angle to another, a target may be illuminated by many pulses in one CPI in the very short dwell time. Therefore, the received echoes are spatially coherent, and additional pulse information about the target may be acquired with the observed pulses. By exploiting this spatial continuity information, we can try to extrapolate or predict the echo information outside the observed CPI.

In order to demonstrate the assumption that the echoed signal is continuous in the spatial space with a short dwell time, two slow moving targets modeled with Swerling I [1] are injected into the real airborne radar data set. The Doppler frequencies of the two targets are 195 Hz and 215 Hz, respectively. The azimuth angle of the antenna is corresponding to 40 degree. The airborne radar parameters are listed in Table 1.

**Figure 3:**
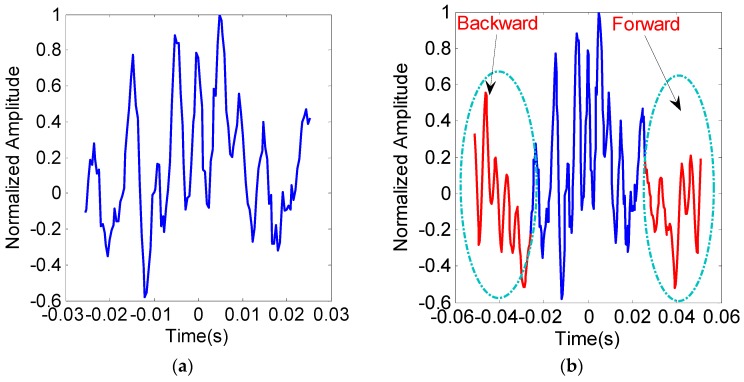

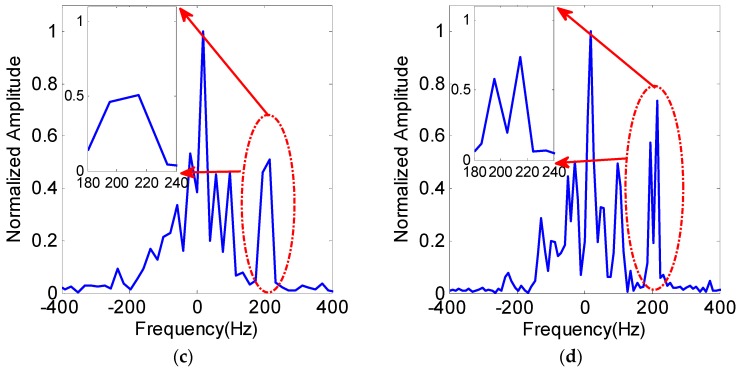
The original signal and the newly merged signal of one range cell in the slow time and the spectrum domain (**a**) Original echoed signal in the slow time domain; (**b**) The newly merged (predicted signal plus original signal) signal based on the spatial continuity property; (**c**)Spectrum of the original echoed signal (**d**); Spectrum of the newly merged signal.

The injected signal just exist about 0.05 s in the slow time domain, which corresponds to about 128 pulses in one CPI with the given dwell time. We predict the forward and backward pulse by exploiting the observed echo information. Detailed forward and backward prediction algorithm will be given in the Section 3.2. The echoed signal of one range cell in the slow time domain is shown in Figure 3.

Based on the spatial continuity assumption, the predicted forward and backward echo information is colored in red, while the initial echo pulse in blue. Detailed pulse information prediction method will be introduced in the Section 3.2, and the prediction factor is set as 0.5 in the experiment. Because the forward and backward pulse lengths are half of the length of one CPI, the equivalent CPI length (i.e., about 0.1 s) in the newly merged signal is doubled than the original CPI length (i.e., about 0.05 s) as shown in Figure 3b. Performing the Fourier analysis to these two data sets shown in Figure 3a,b, respectively.

The corresponding spectrum information can be found in Figure 3c,d, respectively. The zoomed in spectrum information about this two targets is left-top of the Figure. Since the Doppler frequency difference between the two targets is 20 Hz, conventional FFT method cannot distinguish them in the spectrum. The spectrum of the two targets is aliasing together and has just one peak in Figure 3c. However, by exploiting the prior knowledge of the spatial continuity, we can distinguish them well in the frequency domain as shown in Figure 3d. This experiment demonstrates that the assumption that the echoed signal is spatially continuous in the spatial space, and the cross-range resolution can be increased if the spatial continuity property is well exploited in DBS imaging.

### 3.2. KA-DBS Imaging Algorithm

From above analysis, it can be known that the Doppler resolution is proportional to the CPI $T_{a}$ in one azimuth angle. Therefore, we emphasize on increasing the equivalent number of pulses in each CPI by exploiting the spatial continuity information.

By exploiting the spatial continuity information, the forward pulses and the backward pulses outside the measured CPI can be predicated by taking the advantage of the autoregressive (AR) technique [20,21]. AR is a technique that can forecast the future values on the basis of past values of a time series data, which has well been used in ISAR imaging [22]. In this section, we will introduce the AR technique into the DBS imaging. Firstly, the spatial continuity model of the echoed signal should be constructed. Suppose range compression is performed, the forward and backward predicated pulses can be expressed as:

(17) $$\begin{array}{cc} s^{f}\left(\tau ,n\right) & =-\sum_{i=1}^{P}a_{P}\left(i\right)\cdot \sigma \cdot \mathrm{rect}\left(\frac{\left(n-i\right)\cdot t_{r}}{T_{a}}\right)\\ & \cdot \sin c\left[B\left(\tau -\frac{2R\left(\left(n-i\right)t_{r}\right)}{c}\right)\right]\exp\left(-j4\pi \frac{R\left(\left(n-i\right)t_{r}\right)}{\lambda }\right) \end{array}$$

(18) $$\begin{array}{cc} s^{b}\left(\tau ,n-P\right) & =-\sum_{i=1}^{P}a_{P}^{H}\left(i\right)\cdot \sigma \cdot \mathrm{rect}\left(\frac{\left(n-P+i\right)\cdot t_{r}}{T_{a}}\right)\\ & \cdot \sin c\left[B\left(\tau -\frac{2R\left(\left(n-P+i\right)t_{r}\right)}{c}\right)\right]\exp\left(-j4\pi \frac{R\left(\left(n-P+i\right)t_{r}\right)}{\lambda }\right) \end{array}$$

Since our emphasis is on how to improve the cross-range resolution. For clear expression, Equations (17) and (18) can be simplified as:

(19) $$s^{f}\left(\tau ,n\right)=-\sum_{i=1}^{P}a_{P}\left(i\right)s\left(\tau ,n-i\right)$$

(20) $$s^{b}\left(\tau ,n-p\right)=-\sum_{i=1}^{P}a_{P}^{H}\left(i\right)s\left(\tau ,n-p+i\right)$$

Then, the forward and backward prediction error of the echoed signal can be calculated as:

(21) $$e^{f}\left(\tau ,n\right)=s^{f}\left(\tau ,n\right)+\sum_{i=1}^{P}a_{P}\left(i\right)s\left(\tau ,n-i\right)$$

(22) $$e^{b}\left(\tau ,n-p\right)=s^{b}\left(\tau ,n-p\right)+\sum_{i=1}^{P}a_{P}^{H}\left(i\right)s\left(\tau ,n-p+i\right)$$

where *s^f^*(*τ,n*) and *s^b^*(*τ,n–p*) denote the forward and backward prediction pulse, respectively, and *e^f^*(*τ,n*) and e*s^b^*(*τ,n–p*) denote the forward and backward prediction error, respectively. *a*(*i*) represents the AR model coefficient. *P* is the AR model order, and [·]^H^ denotes the conjugate transpose.

In order to obtain the AR model coefficient *a*(*k*), the criterion that to minimize the sum of the forward and backward prediction errors in each iterative procedure is utilized [20,22], which is given as:

(23) $$E_{P}=\;\frac{\sum_{n=P+1}^{N}{\left|e^{f}\left(\tau ,n\right)\right|}^{2}+{\left|e^{b}\left(\tau ,n\right)\right|}^{2}}{2}$$

To solve Equation (23), the Levinson recursion algorithm [24,25,26] is used, which can be solved as follows:

(24) $$a_{P}\left(i\right)=a_{P-1}(i)+a_{P}(P)a_{P-1}^{H}(P-i),i=1,2,\ldots ,P-1$$

By substituting Equation (24) into Equation (19) and Equation (20), respectively, we can have:

(25) $$e_{P}^{f}\left(\tau ,n\right)=e_{P-1}^{f}\left(\tau ,n\right)+a_{P}(p)e_{P-1}^{b}\left(\tau ,n-1\right)$$

(26) $$e_{P}^{b}\left(\tau ,n\right)=e_{P-1}^{b}\left(\tau ,n-1\right)+a_{P}^{H}(p)e_{P-1}^{f}\left(\tau ,n\right)$$

Since the forward and backward predictions are known in each iteration, then the AR coefficients *a_p_*(*p*) can be calculated as:

(27) $$a_{p}(p)=\frac{-2\sum_{n=P+1}^{N}e_{p-1}^{f}\left(\tau ,n\right){\left(e_{p-1}^{b}\left(\tau ,n-1\right)\right)}^{H}}{\sum_{n=P+1}^{N}{\left|e_{p-1}^{f}\left(\tau ,n\right)\right|}^{2}+{\left|e_{p-1}^{b}\left(\tau ,n-1\right)\right|}^{2}}$$

**Figure 4:**
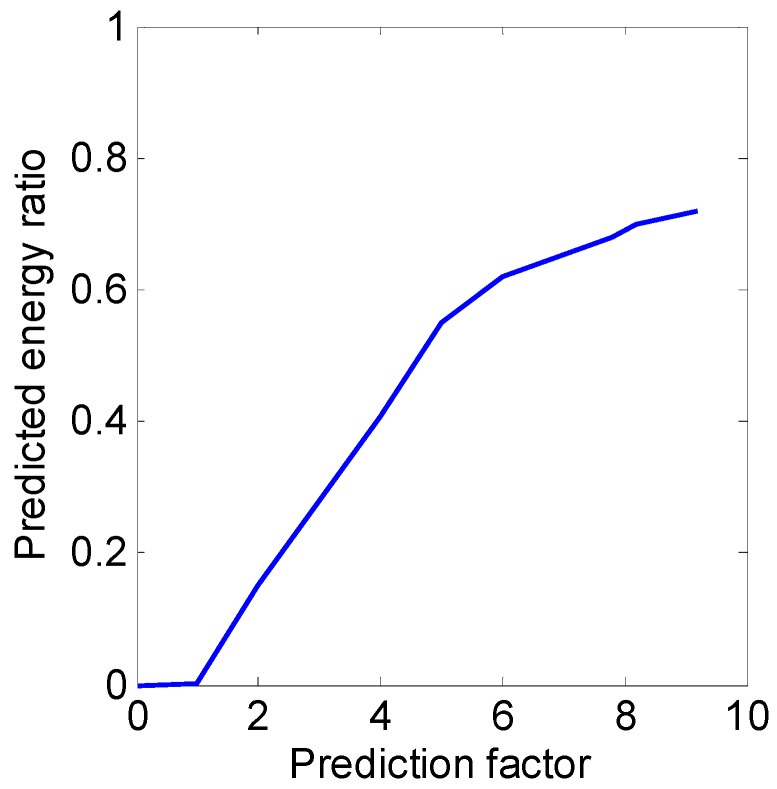
Predicted energy ratio curve with the prediction factor.

Based on the calculated AR coefficients *a_p_*(*p*),, the forward and the backward signals (i.e., *s^f^*(*τ,n*) and *s^b^*(*τ,n–p*) and can be calculated as:

(28) $$\begin{array}{cc} s^{f}\left(\tau ,n\right) & =-\sum_{i=1}^{P}a_{P}\left(i\right)\cdot \sum_{k=1}^{K}\sigma_{k}\cdot \mathrm{rect}\left(\frac{\left(n-i\right)\cdot t_{r}}{T_{a}}\right)\\ & \cdot \sin c\left[B\left(\tau -\frac{2R\left(\left(n-i\right)t_{r}\right)}{c}\right)\right]\exp\left(-j4\pi \frac{R\left(\left(n-i\right)t_{r}\right)}{\lambda }\right) \end{array}$$

(29) $$\begin{array}{cc} s^{b}\left(\tau ,n-P\right) & =-\sum_{i=1}^{P}a_{P}^{H}\left(i\right)\cdot \sum_{k=1}^{K}\sigma_{k}\cdot \mathrm{rect}\left(\frac{\left(n-P+i\right)\cdot t_{r}}{T_{a}}\right)\\ & \cdot \sin c\left[B\left(\tau -\frac{2R\left(\left(n-P+i\right)t_{r}\right)}{c}\right)\right]\exp\left(-j4\pi \frac{R\left(\left(n-P+i\right)t_{r}\right)}{\lambda }\right) \end{array}$$

where *K* is the number of scatters in one range cell. In order to fully utilize the forward and the backward signal, we will merge them with the original signal in the azimuth direction (i.e., the slow time domain). Then the newly merged signal in one range cell can be expressed as:

(30) $$\hat{s}\left(\tau ,1:N+2P\right)=\left[s^{b}\left(\tau ,N-P\right),\ldots ,s^{b}\left(\tau ,N-1\right),s\left(\tau ,1\right)\ldots ,s\left(\tau ,N\right),s^{f}\left(\tau ,N+1\right),\ldots ,s^{f}\left(\tau ,N+P\right)\right]$$

where $\hat{s}\left(\tau ,1:N+2P\right)$ is the newly merged signal in the azimuth direction. One important problem is how to determine the order of the AR model. Shall we increase P infinitely if we want to acquire more predicted signal? Of course not. The selection criteria of the AR model order is detailed discussed in [25,26], and the AR model order is set as one third of the data length in our experiment. Figure 4 gives the predicted energy ratio curve with the predictor factor. In Figure 4, the predicted energy ratio is defined as the energy amount of the predicted signal to the energy amount of the range profile.

From Figure 4, we can know that a higher predictor factor corresponding to a higher predicted energy ratio, which can be explained that more predicted signal contribute more energy. Higher energy radio means a higher uncertainty. Typically, predictor factors less than 3 always lead to useful results (detailed information can be found in [24]). Therefore, the predictor factor is set as 0.5 in our proposed KA-DBS algorithm.

### 3.3. Performance of the Cross-Range Resolution in KA-DBS

Assuming that the prediction factor is 0.5 in the proposed KA-DBS, then the CPI length in each azimuth angle can be estimated as:

(31) $${\hat{T}}_{a}=2NT_{r}=2T_{a}$$

Now, the Doppler resolution in the proposed KA-DBS method is approximately given as:

(32) $$\delta {\hat{f}}_{d}=\frac{1}{{\hat{T}}_{a}}=\frac{\delta f_{d}}{2}$$

Since the equivalent number of pulse (i.e., the CPI length) is doubled in the proposed KA-DBS imaging algorithm, a finer Doppler frequency resolution can be achieved. Therefore, the sharpening ratio in KA-DBS is given as:

(33) $${\hat{K}}_{a}=\Delta f_{d}/\delta {\hat{f}}_{d}=2K_{a}$$

As illustrated in Equation (33), the sharpening ratio ${\hat{K}}_{a}$ is theoretically improved by a factor of 2 compared to the conventional DBS imaging algorithm. Therefore, a high cross-range resolution and large sharpening ratio can be achieved in the proposed KA-DBS imaging framework.

### 3.4. Super-Resolution Imaging Algorithm Based on KA-DBS

**Figure 5:**
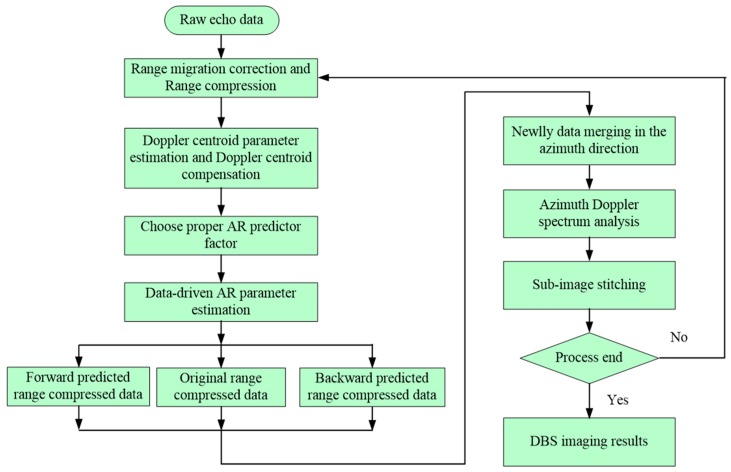
Process of the proposed KA-DBS processing approach.

So far, the implementation of the proposed KA-DBS super-resolution imaging approach for WAS radar has been described. Figure 5 below shows the whole imaging procedure.

In Figure 5, first, range migration corrections and range compression are utilized to process the raw echo data. Second, the Doppler centroid estimation parameter is estimated, and the Doppler centre of the signal is modulated to zero frequency, which is quite useful for the latter sub-image stitching process. Third, the proper AR predictor factor is set, and the AR parameter can be data-driven based on the Levinson recursion algorithm. Fourth, the forward range compressed data prediction and the backward range compressed data prediction are performed simultaneously. Then, the forward predicted range compressed data, the backward predicted range compressed data and the original range compressed data are merged together to form a newly merged data. After that, the Doppler analysis is performed to form the DBS sub-image. Finally, the final fan DBS image is formed by stitching all the DBS sub-images based on the affine transformation [9]. The comparison of different super-resolution methods is demonstrated in the following section.

## 4. Experimental Results

### 4.1. Simulation

**Figure 6:**
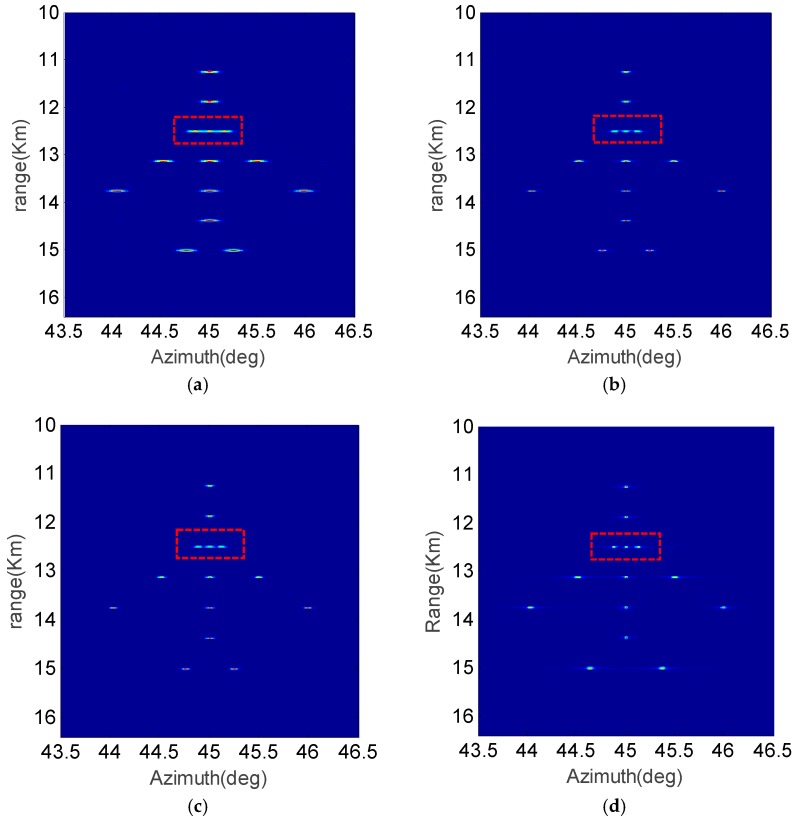
Simulation results in the case of SNR=10 dB; (**a**) FFT algorithm; (**b**) Relax algorithm; (**c**) APES algorithm; (**d**) KA-DBS algorithm.

The point target simulations are performed in Figure 6. The point targets are distributed as the outline of an airplane. The simulation parameters are illustrated in Table 1.

Figure 6 shows the imaging results with FFT, Relax, APES and the proposed KA-DBS algorithm. The SNR is set as 10 dB. From Figure 6, it can be seen that the imaging results based on the FFT algorithm is a little blurred, especially for the closely spaced scatters in the red rectangle, while the closely spaced scatters can be well distinguished in the imaging results based on Relax, APES and the proposed KA-DBS algorithm. Moreover, the image generated by the proposed KA-DBS algorithm is much clear than that of the other algorithms.

**Figure 7:**
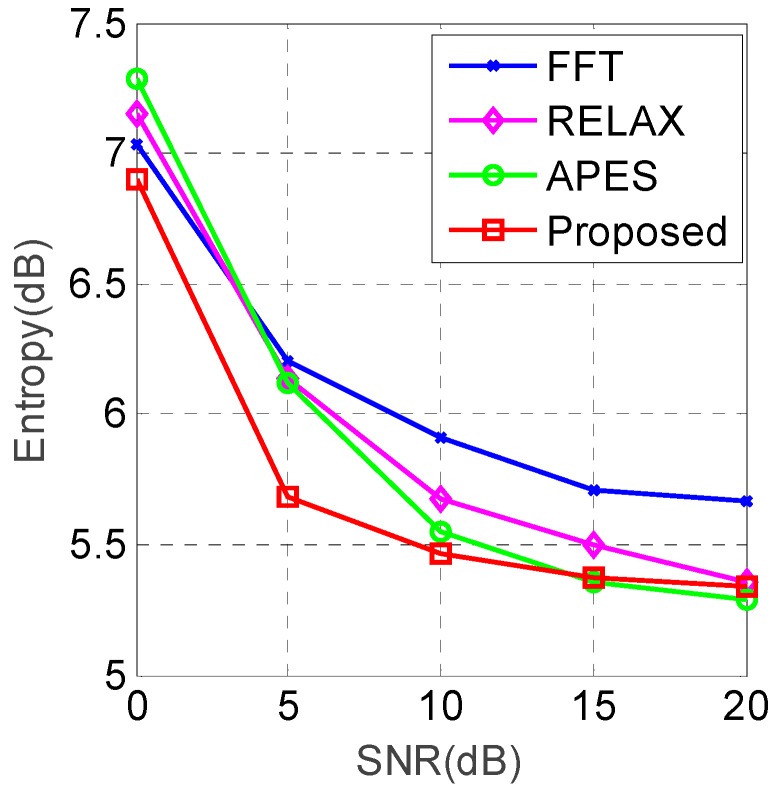
Entropy curves with different algorithms.

The entropy curve of different imaging algorithms under different SNRs is shown in Figure 7.

From Figure 7, it can be seen that the entropies of all the algorithms are decreasing with the increase of SNR. The proposed algorithm has the smallest entropy compared with the other algorithms when the SNR is less than 15 dB, which is in accordance with the imaging results in Figure 6. An interesting phenomenon can be seen form Figure 7 that the entropy of the proposed algorithm is a little higher than that of the APES algorithm when the SNR is higher than 15 dB. This may be explained that the APES algorithm has lower sidelobes than the proposed algorithm, and the lower sidelobes contribute more in the process of entropy computing under the high SNR situations for the simple simulation scene. The simulation results demonstrate that the proposed KA-DBS imaging algorithm outperforms the other algorithms.

### 4.2. Real Data

We study the performance of the newly proposed KA-DBS algorithm by resorting to real data set in this section. The experimental data collected on the wide-area surveillance mode of an airborne radar system is selected. The experiment radar parameters are illustrated in Table 2.

**Figure 8:**
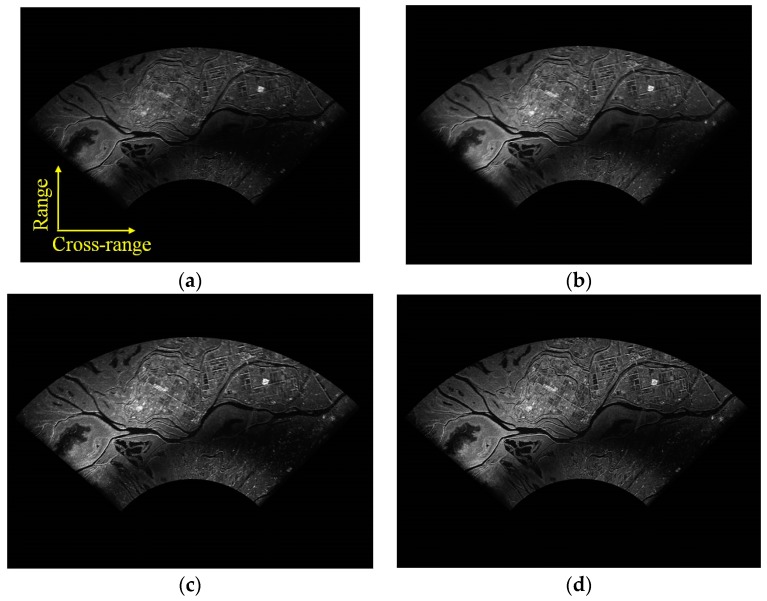
Imaging fan results; (**a**) FFT algorithm with 128 pulses; (**b**) Relax algorithm with 128 pulses; (**c**) APES algorithm with 128 pulses; (**d**) KA-DBS algorithm with 128 pulses.

**Figure 9:**
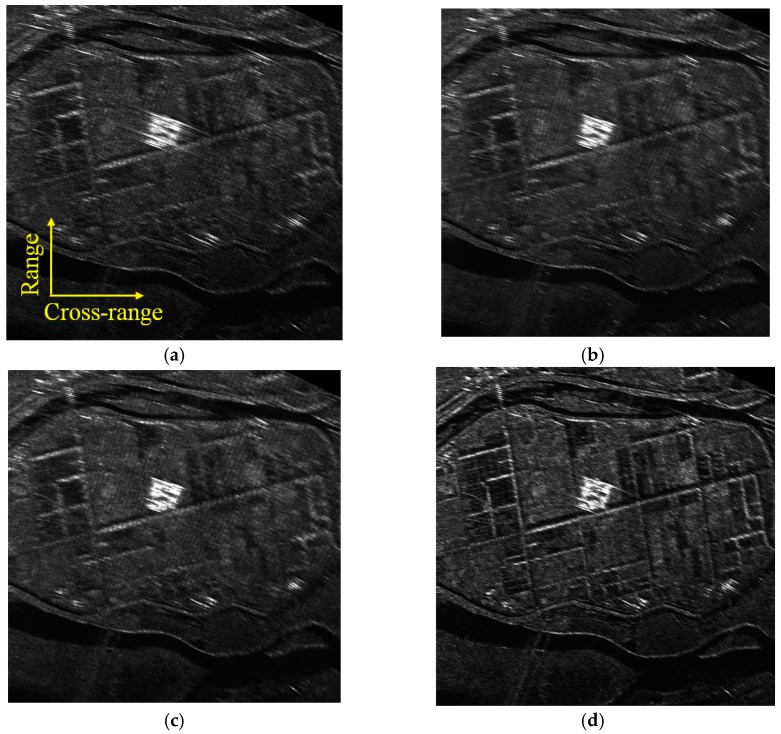
Locally imaging results; (**a**) FFT algorithm with 128 pulses; (**b**) Relax algorithm with 128 pulses; (**c**) APES algorithm with 128 pulses; (**d**) KA-DBS algorithm with 128 pulses.

The DBS imaging results using different algorithms (i.e., the conventional FFT-based, Relax-based, APES-based and the proposed KA-DBS algorithm) are given in Figure 8. In KA-DBS, the predicted forward and backward pulse number is half of the pulse number in one CPI. The SNR is about 18 dB in the experiment. All the sub-images are stitched together based on the affine transformation algorithms [9]. In Figure 8a, the image in the conventional FFT-based algorithm suffers from blur. Moreover, it is obvious that the imaging results become clear and clear from the upper-left part of Figure 8 to the lower-right part of Figure 8. And the imaging result based on KA-DBS in Figure 8d performs the best. To further analyze the imaging results, Figure 9 shows the same zoomed in patch of Figure 8.

From Figure 9, it can be found that the proposed KA-DBS algorithm can focus the scene much clear than the other algorithms, and the texture information can be easily distinguished. For example, the roads in Figure 9d are much thinner than that in Figure 9a–c. This can be explained that the forward and backward predicted pulses in each CPI are well utilized to improve the cross-range resolution in KA-DBS imaging.

**Figure 10:**
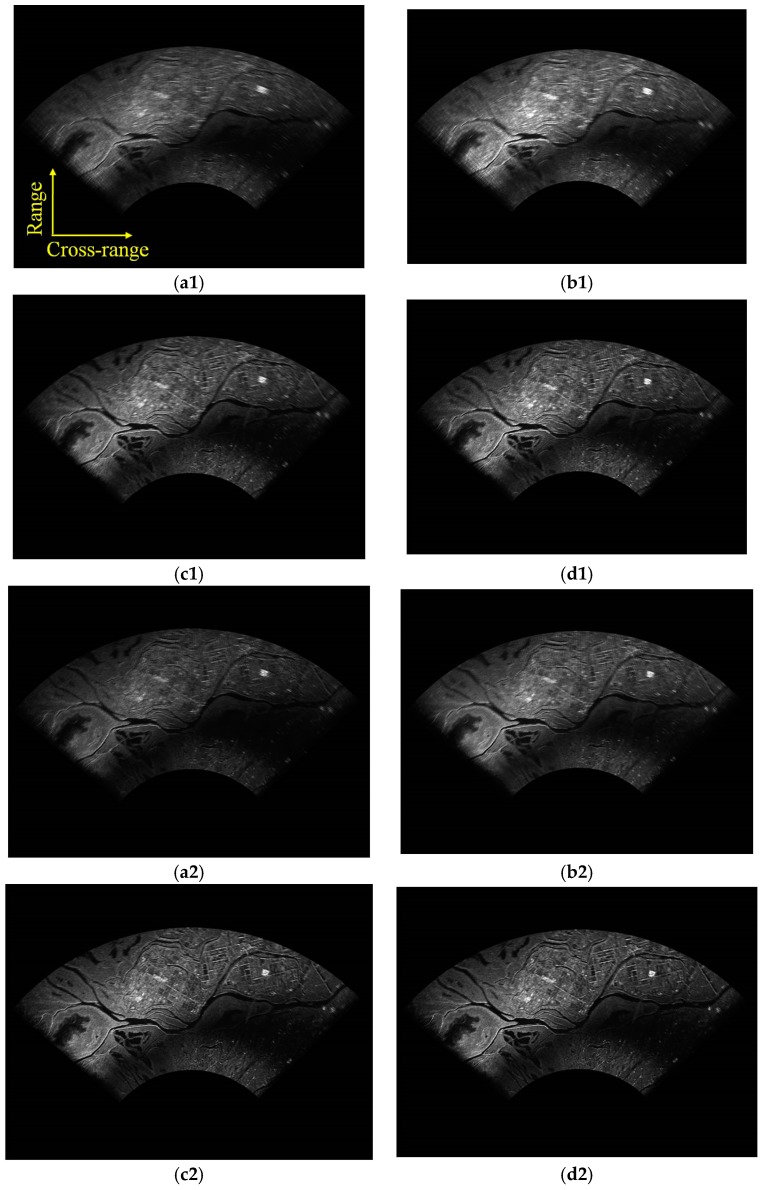
Imaging fan results; (**a1**) FFT algorithm with 32 pulses; (**b1**) Relax algorithm with 32 pulses; (**c1**) APES algorithm with 32 pulses; (**d1**) KA-DBS algorithm with 32 pulses; (**a2**) FFT algorithm with 64 pulses; (**b2**) Relax algorithm with 64 pulses; (**c2**) APES algorithm with 64 pulses; (**d2**) KA-DBS algorithm with 64 pulses.

In order to further verify the proposed algorithm, the coherent pulse number is changed from 32 to 64, and the selected pulse is the central part of the echoed signal. For example, the 49th to the 80th pulse in each azimuth angle is selected to form the 32 pulses data set, and the 33rd pulse to the 96th pulse in each azimuth angle is selected to form the 64 pulses data set. Different algorithms with different pulses are compared in Figure 10. Figure 10a1–d1 are the imaging results with 32 pulses, and Figure 10a2–d2 are results with 64 pulses. From the upper-left to the lower-right, FFT-based, Relax-based, APES-based and the proposed KA-DBS algorithm are given. It can be seen that the imaging results become better and better from the upper-left part of Figure 10 to the lower-right part of Figure 10.

**Figure 11:**
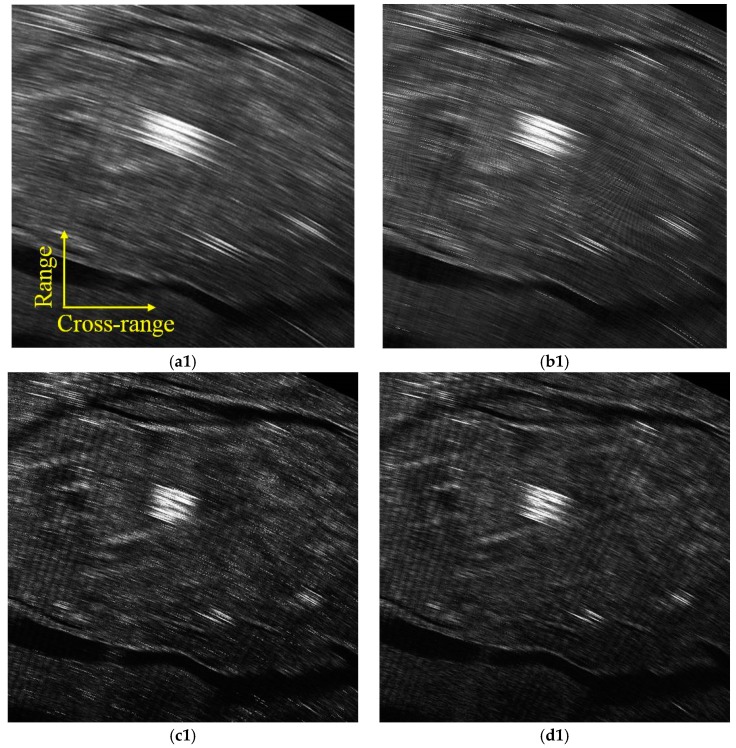

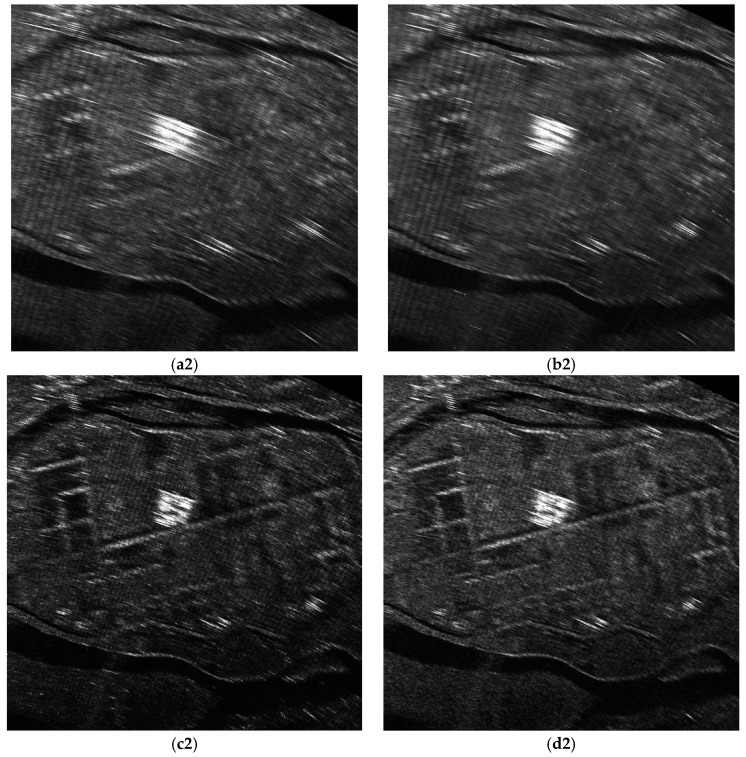
Locally imaging results; (**a1**) FFT algorithm with 32 pulses; (**b1**) Relax algorithm with 32 pulses; (**c1**) APES algorithm with 32 pulses; (**d1**) KA-DBS algorithm with 32 pulses; (**a2**) FFT algorithm with 64 pulses; (**b2**) Relax algorithm with 64 pulses; (**c2**) APES algorithm with 64 pulses; (**d2**) KA-DBS algorithm with 64 pulses.

The zoomed in patch of the same zone in Figure 10 is shown in Figure 11. From Figure 11, we can see that the imaging results with 64 pulses are much clear than that with 32 pulses for the same imaging algorithm. Moreover, the image based on FFT method under 32 pulses is the most blurred while the image based on the proposed KA-DBS method under 64 is the most clear. The road, farmland and other detailed information can be well distinguished in the proposed KA-DBS imaging results. Therefore, the proposed KA-DBS algorithm can improve the cross-range resolution at the situation of short dwell time.

## 5. Discussion

In this part, we will evaluate the imaging performance of the KA-DBS algorithm with the other imaging algorithms under different CPI lengths. To estimate the imaging quality, entropy is always utilized, which can be defined as [8]:

(34) $$E=-\sum_{m=1}^{M}\sum_{n=1}^{N}p_{m,n}\log\left(p_{m,n}\right)$$

where the probability distribution function is:

(35) $$p_{m,n}=\frac{I_{m,n}^{2}}{\sum_{m=1}^{M}\sum_{n=1}^{N}I_{m,n}^{2}}$$

In Equation (35), *I_m,n_* (*m* = 1,2,…,*M, n* = 1,2,…,*N*) is the concerned image, which is an *M*×*N* matrix.

**Figure 12:**
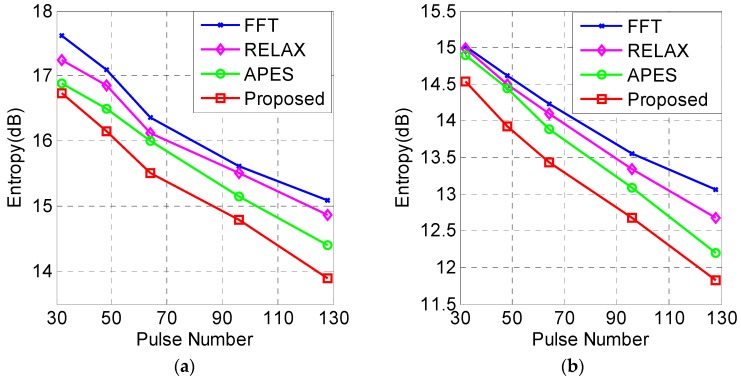
Entropy curves with different algorithms (**a**) the fan images (**b**) the local images.

The entropy curves of the wide-area image and the local image are shown in Figure 12a,b, respectively.

From Figure 12, we can find that the entropy of the KA-DBS algorithm is the lowest, which means that a higher cross-range resolution can be acquired. Based on above experimental results, it proves that the proposed KA-DBS algorithm outperforms the other algorithms.

## 6. Conclusions

In this paper, we have considered the problem of high cross-range resolution DBS imaging for airborne WAS radar in short dwell time situations. A knowledge-aided Doppler beam sharpening (i.e., KA-DBS) imaging algorithm is proposed. We have investigated the spatial property of the echoed signal, and the spatial continuity model of the airborne radar system is constructed. By exploiting this spatial continuity knowledge, the forward and backward pulse information outside the observed CPI is well predicted based on the AR technique. Then the predicted pulses are merged together with the original pulses to form the newly merged pulses. Finally, DBS imaging is performed. The number of newly merged pulses in the proposed KA-DBS algorithm is twice larger than that in the conventional DBS algorithm with the same dwell time. Therefore, the cross-range resolution is improved by a factor of two in KA-DBS. Real airborne experiments have demonstrated that the proposed KA-DBS algorithm performs well with short dwell time.

## Figures and Tables

**Table 1:** Radar System Parameter.

Parameters	Value
Time width	10 s
Band width	12 MHz
Pulse repetition frequency	2500 Hz
Azimuth beam width	3.2°
Coherent pulses	128
Range gate number	2048

**Table 2:** Radar System Parameter.

Parameters	Value
Time width	24 us
Band width	40 MHz
Pulse repetition frequency	2500 Hz
Scanning area	45°~135°
Coherent pulses	128
Range gate number	4096
